# Comparison of smartwatch-derived energy expenditure estimation during outdoor activities

**DOI:** 10.3389/fdgth.2026.1926837

**Published:** 2026-08-24

**Authors:** Haedo Cho, Chelsey Campillo Rodriguez, Patrick Slade

**Affiliations:** 1 John A. Paulson School of Engineering and Applied Sciences, Harvard University, Boston, MA, United States; 2 Kempner Institute for the Study of Artificial and Natural Intelligence, Harvard University, Boston, MA, United States

**Keywords:** energy expenditure, heart rate, mobile health, smartwatch, validation

## Abstract

Smartwatches are commonly used to estimate energy expenditure, but studies have reported errors of 25% to 70%, possibly due to factors such as activity type, use case, and sensor data. We compared three readily available configurations for estimating energy expenditure during outdoor walking and running: a smartwatch operated in its activity-specific mode that incorporated GPS, IMU, and heart rate data; a smartwatch operated in its generic, non-activity-specific mode that incorporated IMU and heart rate data; and a pre-defined heart rate model. Thirty adults (17 men, 13 women; 34 ± 14 years) completed outdoor activities while wearing a portable respirometry system and both smartwatches. Participants performed self-paced walking and running conditions, and followed ecologically relevant audio prompts to elicit real-world walking. Cumulative energy expenditure from each method was compared with ground-truth respirometry. The activity-specific smartwatch achieved 13% error during outdoor walking with 2.2–2.8 times lower error than the generic smartwatch and heart rate model, which could be attributed to its activity-specific algorithm, GPS availability, hardware, or a combination of these factors. The heart rate model yielded a lower error than both the activity-specific and non-activity-specific smartwatches during outdoor running in this cohort. This study reflects practical differences between commercially available setups as device, sensor suite, and proprietary algorithms differ across these tools. Estimates from non-activity-specific smartwatches, such as the Fitbit Charge 4 tested here, should be interpreted cautiously by researchers, clinicians, and users when monitoring physical activity.

## 1 Introduction

Smartwatches show promise for monitoring many health metrics, but currently do not meet lab-grade standards for physical activity monitoring. Equipped with a suite of sensors, smartwatches can monitor a range of physiological data such as heart rate, physical activity, sleep pattern, and stress levels (1). Several smartwatches have received medical-grade clearance for cardiovascular health monitoring such as atrial fibrillation detection, indicating a promising application for clinical research (1). Health organizations have requested physical activity monitoring tools that use wearable sensors to provide objective metrics like energy expenditure (2) to replace subjective self-report surveys that introduce substantial error (3). However, laboratory validation studies have reported smartwatch energy expenditure estimation errors ranging from 25% to 70% (4–7), which exceeds the 10% threshold considered acceptable for free-living condition studies (8). Challenges monitoring lower-limb activities such as walking, running and biking may contribute to these errors due to differences between leg and wrist motion (9). Lower-limb activities account for a larger portion of daily energy expenditure than upper-limb activities, making accurate monitoring of these movements critical.

Smartwatch accuracy for estimating energy expenditure may depend on the use case: the activity, indoor or outdoor use, and whether an activity-specific mode was selected by the user. Estimation accuracy depends on activity type (10), with some activities being more challenging to capture the relationship between smartwatch data and energy expenditure. The use case impacts performance by determining which smartwatch sensors and data-driven model are used to estimate energy expenditure. Smartwatch sensors may include a triaxial inertial measurement unit (IMU) that captures wrist motion, a heart rate sensor that captures a physiological measure of exertion, and a global positioning system (GPS) that captures pace and distance during outdoor activities. These sensors provide different physiological and movement data related to energy expenditure, but it is unclear which sensors are most informative. Indoor or outdoor activity impacts which smartwatch sensors are available. Sensor data are subsequently passed into a proprietary data-driven model that estimates energy expenditure, with different smartwatch brands having different models and performance (4, 10). Using an activity-specific mode selected by the user, such as walking or running (11), may utilize different data-driven models that could improve energy expenditure estimation by leveraging additional constraints or interpretations of motion for that specific activity.

**Figure 1:**
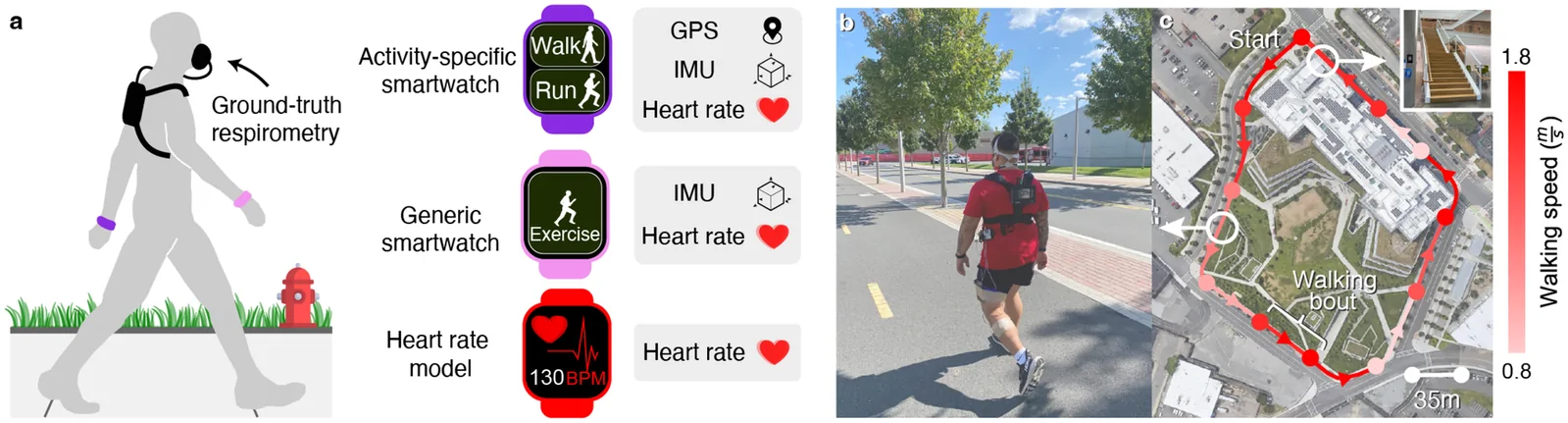
Experimental setup for evaluating smartwatch energy expenditure estimation during outdoor activities. **(a)** Participants performed physical activity experiments in a community setting while wearing an activity-specific smartwatch, a generic smartwatch, and a portable respirometry system for ground-truth measurement of metabolic cost. **(b)** Example of a participant performing the experiment on a public sidewalk. **(c)** Map of the 650-meter course used for evaluation. Participants performed 20 bouts of walking through the course while following ecologically relevant audio prompts to elicit naturalistic walking. Each lap included two flights of indoor stair climbing.

This study evaluated how well different smartwatch use cases estimated energy expenditure for common aerobic activities like walking and running. The data-driven energy expenditure models used by smartwatches are not open-source, obscuring which sensor data are utilized and the underlying model structure. This study was designed to characterize how different commercially-available configurations impacted energy expenditure estimation during outdoor activities for: a smartwatch operated in an activity-specific mode that incorporated GPS, IMU, and heart rate data; a smartwatch operated in a generic, non-activity-specific, mode that incorporated IMU and heart rate data; and a heart rate model (Figure 1a). Energy expenditure estimates were collected from each smartwatch's corresponding smartphone application, while a predefined heart rate model (12) was used to isolate a model that did not take into account wrist motion. The performance of the activity-specific smartwatch during outdoor activities was compared to a prior study using the same smartwatch during indoor activities (11) to investigate whether GPS availability may explain differences in smartwatch monitoring.

## 2 Methods

### 2.1 Study participants

We recruited participants across a range of age and body mass indices (*n* = 30, 17 men and 13 women; age, 34 ± 14 yr; body mass, 71.4 ± 13.0 kg; height, 1.72 ± 0.08 m; body mass index, 24.1 ± 3.5 kg/m^2^) similar to those in a previous study that validated smartwatch performance from a diverse cohort (4). We aimed to recruit participants across diverse cohorts to best replicate the true U.S population, however this cohort may not be fully representative of the general population, including older adults, individuals with obesity, clinical or chronic disease populations, and individuals with atypical gait. We also screened for and recruited a generally active and healthy population, but their fitness may have varied, which could cause potential bias or error in energy expenditure estimates. Participants could skip any conditions they found too strenuous to make the protocol feasible for individuals with diverse physiological characteristics. Five participants chose to skip the outdoor running. Two participants were excluded due to connection issues with the portable respirometry system during real-world walking. Participants provided written consent before the study, and all experiments were approved by the Institutional Review Board of Harvard University (IRB22-1561) and Stanford University (IRB-17282).

### 2.2 Physical activity monitoring tools

Participants wore two smartwatches on either wrist and a portable respirometry system (K5, COSMED) for measuring ground-truth energy expenditure (Figure 1a). The portable respirometry calculated ground-truth metabolic cost of physical activities using measured volume of carbon dioxide and oxygen exchanged with each breath. The standard Brockway equation was applied to convert gas exchange data into metabolic cost in Watts for each breath (11). The activity-specific smartwatch was an Apple Watch Series 1 (42 mm) with model number A1154 and software version 4.3.2. GPS movement trajectory was collected from a paired smartphone, iPhone 12 Pro with a model number of MGK13LL/A. Participants selected the activity type on the Apple Watch prior to each activity, such as *outdoor walk* or *outdoor run*. While there are newer Apple Watch models, the Series 1 model contains the same type of core sensors as updated models and uses the same smartphone application to estimate energy expenditure. Due to the model's black-box nature, it is unclear whether newer smartwatches produce different energy expenditure estimates. The generic smartwatch was the Fitbit Charge 4 with model number FB417 and software version 48.20001.100.76. This smartwatch estimates energy expenditure during activities without being configured for any activity. Both smartwatches were calibrated to each individual by entering subject-specific information including height, weight, and sex in the smartwatch application. The heart rate-based model estimated energy expenditure every 5 s using a regression equation that incorporates heart rate collected by the activity-specific smartwatch, and the subject's sex, age, height, and weight (12).

### 2.3 Real-world study protocol

Participants walked or ran on a public sidewalk wearing a portable respirometry system, an activity-specific smartwatch on the right wrist (Apple Watch), and a generic smartwatch on the left wrist (Fitbit) (Figures 1a,b). These smartwatches were selected because they had the lowest error among smartwatch brands in previous studies (4, 6, 10). Participants performed 3 different outdoor, overground activities: self-paced 6 min of walking, self-paced 6 min of running, and naturalistic real-world walking consisting of 20 different bouts at varying self-selected speeds (Figure 1c; Supplementary Table S1). Cumulative energy expenditure was compared between ground-truth respirometry and the smartwatch models.

To elicit naturalistic walking that vary in speed, participants performed 20 walking bouts at various durations guided by audio prompts (e.g., ‘walk as if you were walking across the street’ or ‘walk as if you had a really bad day’). These prompts are based on a prior study demonstrating that they produce a broad range of self-selected speeds representative of real-world walking (13, 14). Participants rested in a quiet standing position for a randomized duration of 5–10 s between each walking bout. All walking and running conditions were completed along a 650-m path that was primarily outdoors, except for two flights of stairs which were indoors. To ensure continuous GPS tracking throughout the route, GPS data were obtained during indoor stair-climbing intervals, which comprised only a brief portion of the route. Participants walked and ran at a self-selected steady pace for all outdoor conditions.

### 2.4 Evaluation metric

We evaluated the performance of all physical activity monitors based on absolute percentage error (APE), which is defined as the absolute value of the percent error between estimated and ground-truth cumulative energy expenditure. As smartwatch companies do not disclose their software algorithms, our analysis only includes energy expenditure values provided by their apps. Cumulative energy expenditure was calculated due to the time-varying conditions tested in this study. Estimating instantaneous energy expenditure during time-varying activities is difficult because mitochondrial dynamics cause a delay between energy expended by muscles and gas exchange measured by respirometry (15). Cumulative energy expenditure was determined by summing all estimates, multiplied by the time difference between consecutive estimates, divided by the total time period of activity. The error of cumulative energy expenditure was reported as APE.

### 2.5 Statistical analysis

All statistical tests were implemented using python (3.10.8) and additional python libraries including scipy (1.10.1) and statsmodels (0.14.1). All data were assessed for normality using the Shapiro–Wilk test. Non-parametric tests were used if the normality test rejected the null hypothesis. We used Kruskal–Wallis test for comparisons involving more than two groups and two-sided Wilcoxon signed-rank test for pairwise comparisons. A *post-hoc* multiple comparison test was performed after assessing the main effect using the Kruskal–Wallis test. Rank-biserial correlation was used to measure effect size for Wilcoxon signed-rank comparisons. For multiple comparison tests, the significance threshold was adjusted using the Bonferroni correction to control for Type I error rate. All statistical tests were set to a significance level of 0.05, adjusted to 0.016 after Bonferroni correction, with significance reported below this threshold. A linear mixed-effects model was used to investigate the effects of subject-specific information on energy expenditure estimation error of the activity-specific smartwatch, generic smartwatch, and heart rate model. Age, sex, and BMI were used as fixed effects, with a random intercept for each participant to account for repeated within-subject measurements across real-world walking, outdoor walking, and outdoor running.

## 3 Results

**Figure 2:**
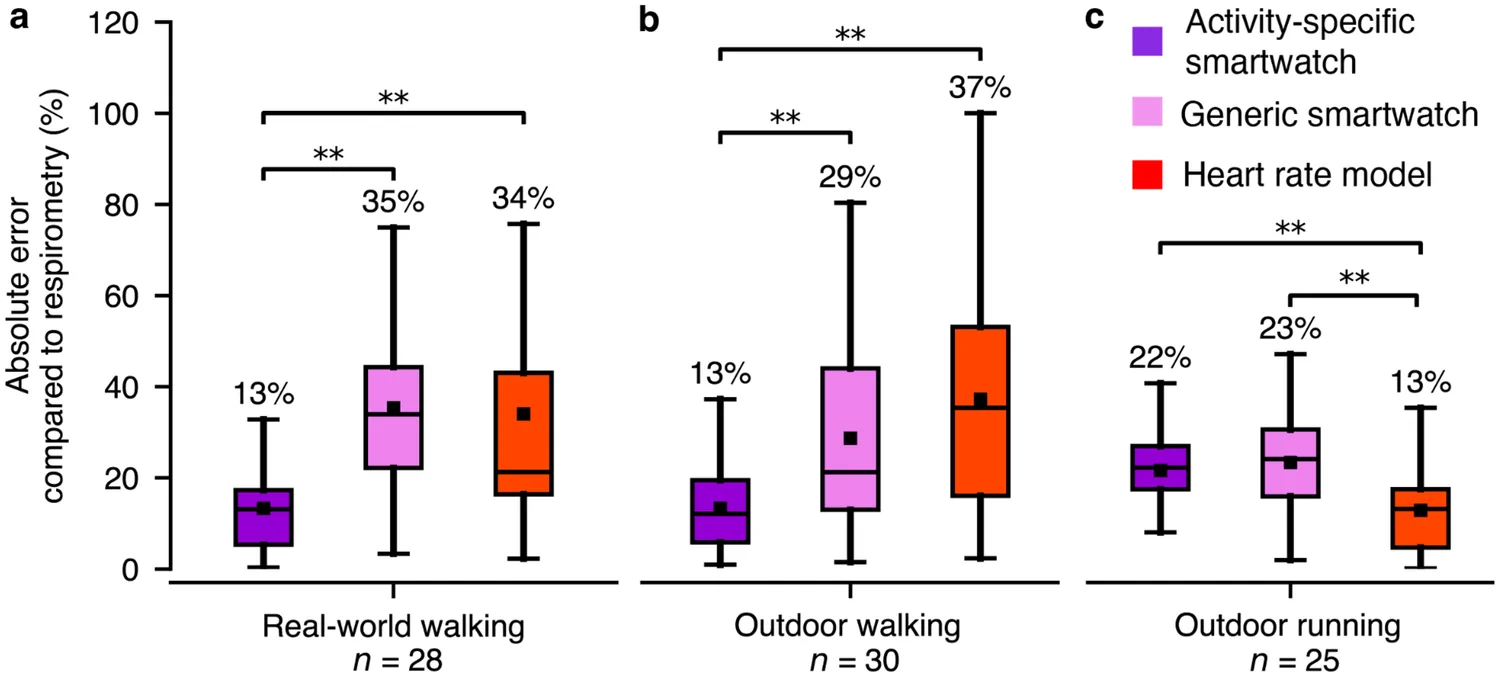
Energy expenditure estimation error for an activity-specific smartwatch, a generic smartwatch, and a heart rate model compared to ground truth respirometry during outdoor activity. The activity-specific smartwatch had a significantly lower absolute percentage error of cumulative energy expenditure during **(a)** naturalistic real-world walking with 20 different bouts and **(b)** outdoor walking at a self-selected constant speed than the generic smartwatch or heart rate model (**P < 0.016). **(c)** The heart rate model had significantly lower error than both smartwatches during outdoor running (**P < 0.016). All pairwise comparisons used Wilcoxon signed-rank tests with Bonferroni correction (ɑ = 0.016); *p*-values and effect sizes are reported in Table 2. The boxes show the interquartile range, with a line at the median and a dot and percentage value representing the mean. Whiskers extend to 1.5 times the interquartile range.

Using a smartwatch in activity-specific mode during outdoor walking performed similarly to recommended activity monitoring guidelines and significantly better than outdoor running in this protocol. The activity-specific smartwatch had an average error of 13% during naturalistic real-world walking and outdoor walking (Table 1; Figures 2a,b), approaching the threshold for laboratory-grade equipment (8). This error is substantially lower than what previous smartwatch studies have reported, with the activity-specific and GPS-enabled configuration approaching laboratory-grade accuracy for this specific outdoor walking. Although, this comparison is limited by differences in population and protocol across studies, as well as black-box outcome metrics of smartwatches. The activity-specific smartwatch achieved 2.7 times lower error than the generic smartwatch and 2.6 times lower error than the heart rate model during real-world walking. During outdoor walking, the activity-specific smartwatch achieved 2.2 times and 2.8 times lower error than the generic smartwatch and heart rate model, respectively (Table 1; Figures 2a,b). The heart rate model had significantly lower error than both the activity-specific smartwatch and the generic smartwatch during outdoor running (Table 1; Figure 2c).

**Table 1:** Descriptive statistics for absolute percentage error of energy expenditure estimates by device and condition.

Condition	Method	*n*	Mean APE (95% CI)	Median APE (IQR)
Real-world walking	Apple Watch	28	13.3% (9.8–16.8%)	13.1% (5.3–17.3%)
	Fitbit	28	35.4% (28.0–42.8%)	34.0% (22.2–44.3%)
	Heart rate model	28	34.0% (23.7–44.3%)	21.3% (16.4–43.1%)
Outdoor walking	Apple Watch	30	13.3% (10.0–16.6%)	12.1% (5.8–19.5%)
	Fitbit	30	28.7% (21.0–36.4%)	21.% (13.0–44.0%)
	Heart rate model	30	37.2% (27.6–46.9%)	35.4% (16.0–53.1%)
Outdoor running	Apple Watch	25	21.6% (17.7–25.6%)	22.2% (17.5–27.0%)
	Fitbit	25	23.4% (18.5–28.2%)	24.1% (15.9–30.6%)
	Heart rate model	25	12.9% (8.7–17.0%)	13.2% (4.7–17.5%)

Mean and median APE compared to ground-truth portable respirometry are reported for each method across outdoor conditions. APE, absolute percentage error; CI, confidence interval; IQR, interquartile range.

**Table 2:** Pairwise comparisons of absolute percentage error between device configurations by condition.

Condition	Reference	Comparison	*n*	Rank-biserial *r*	*p*-value
Real-world walking	Apple Watch	Fitbit	28	−0.970	1.04 × 10^−7^
Real-world walking	Apple Watch	Heart rate model	28	−0.828	3.18 × 10^−5^
Outdoor walking	Apple Watch	Fitbit	30	−0.690	5.55 × 10^−4^
Outdoor walking	Apple Watch	Heart rate model	30	−0.845	9.22 × 10^−6^
Outdoor running	Heart rate model	Apple Watch	25	−0.711	1.15 × 10^−3^
Outdoor running	Heart rate model	Fitbit	25	−0.889	1.51 × 10^−05^

Pairwise comparisons were performed using two-sided Wilcoxon signed-rank tests with Bonferroni correction (ɑ = 0.016). Effect sizes are reported as matched-pairs rank-biserial correlation (*r*). Negative *r* values indicate lower absolute percentage error in the reference method relative to the comparison method. All comparisons were statistically significant after correction.

## 4 Discussion

Activity-specific smartwatches and heart rate models may be an effective monitoring tool for outdoor or indoor running. The heart rate model had a similar or lower error when monitoring high-intensity activities like running compared to the activity-specific smartwatch during indoor (11) and outdoor activities (Figure 2c, Supplementary Table S1). This is a surprising finding as adding more information about the person's wrist IMU data and GPS data increased energy expenditure estimation error. However, only the heart rate model was an existing open-source model (12). A direct comparison would require training the exact same type of data-driven model with each combination of sensor inputs to isolate the impact of different types of input data. The heart rate model performed worse during both indoor (11) and outdoor low-intensity activities like walking (Figures 2a,b). This aligns with prior work showing that heart rate is less useful for estimating energy expenditure during low-intensity activities. During rest and light activity, heart rate values often overlap and cannot be reliably distinguished from one another (16). Combining additional biomechanical data with a smartwatch could improve energy expenditure estimation, such as using a smartphone in a pocket to capture leg motion related to muscle activity (14).

The generic smartwatch reported greater error during outdoor walking and running than the activity-specific smartwatch, although this finding is specific to the devices and software versions used in this study. Activity-specific mode, GPS availability, sensor suite, and energy expenditure model design all differ between the Apple Watch and Fitbit, making these factors confounded. Since the data-driven models are proprietary it is unclear what causes this poor estimation performance. Possible contributing factors include lower sensor sampling rate, lack of GPS data, or a generalized model that cannot leverage activity-specific information. The difference in performance between the activity-specific smartwatch (Apple Watch Series 1) and the generic smartwatch (Fitbit Charge 4) suggests that GPS availability may provide important information for monitoring walking, as pace and distance may capture energy expenditure better than wrist motion or heart rate information. The activity-specific smartwatch had an average error of 13% during outdoor walking (Figure 2b), substantially lower than the 27% error reported when GPS was not used during indoor walking in the same activity-specific mode (11). This study was unable to evaluate a smartwatch in generic mode that included GPS data to determine how much the activity-specific mode may impact performance. However, a prior study found the same activity-specific smartwatch performed worse than a generic smartwatch during time-varying indoor walking speeds, suggesting activity-specific models may not be beneficial for estimating energy expenditure during walking (11).

Lack of access to sensor-level data and black-box algorithms, as well as differences in device sensors and data-driven models, are limitations of this study. Additionally, the activity-specific smartwatch used in this study was an older model, which may impact performance, although this version had medical grade accuracy for raw sensor readings like heart rate (4). This study assumed that the Apple Watch Series 1 utilizes the same algorithm to estimate energy expenditure as newer models since the smartphone application used across Apple Watch devices was kept up to date throughout data collection. However, differences in hardware, firmware, sensor quality, and proprietary algorithms across generations mean these findings may not generalize to newer Apple Watch models. This is a common limitation in studies of commercial smartwatch validation research as smartwatch devices are frequently updated and companies do not disclose their algorithms or how they operate (3). Future work should directly compare energy expenditure estimates across device generations to confirm consistency.

Users trying to accurately track energy expended during physical activity should consider using a smartwatch and configuration similar to the activity-specific device tested in this study, such as one that leverages GPS and activity-specific data, while performing outdoor walking or high-intensity exercise. The activity-specific smartwatch used in this study can monitor outdoor walking with a performance approaching laboratory-grade methods, providing a portable method for high-quality physical activity monitoring in a specific use case. Heart rate models may be a reliable tool for users who perform high-intensity training, specifically running, outdoors or indoors. Additional research exploring how smartwatches and other activity monitors perform during a variety of exercises and use cases could better inform monitoring accuracy for many health and science fields. These findings provide insight into how smartwatches, like the Apple Watch Series 1 in activity-specific mode, could enable users, researchers, and clinicians to improve activity monitoring accuracy for personal exercise tracking, research studies, or health planning.

## Data Availability

The datasets presented in this study can be found in online repositories. The names of the repository/repositories and accession number(s) can be found below: https://github.com/Harvard-Slade-Lab/Activity-specific-tracking-estimates-energy-expenditure-with-near-lab-grade-performance-outdoors.
