# Supplementary material for: Comparison of smartwatch-derived energy expenditure estimation during outdoor activities
Source: Front Digit Health. 2026 Aug 24;8:1926837. doi: 10.3389/fdgth.2026.1926837 (PMC13547467; doi:10.3389/fdgth.2026.1926837)

**Supplementary materials**

**Supplementary Table 1.** Comparison of indoor and outdoor study characteristics.

| Study | Study characteristic | Target activities | Activity monitor (device) | Sample size (sex) and demographics |
|---|---|---|---|---|
| Indoor experiment (11) | A diverse group of healthy adults performed steady-state activities under indoor laboratory conditions. | Walking on the treadmill at fixed speeds of 1.0 m/s and 1.5 m/s.<br>Running on the treadmill at fixed speeds of 2.0 m/s and 2.5 m/s. | Activity-specific smartwatch (Apple Watch Series 1, activity specific mode)<br>Smartwatch (Apple Watch Series 1, general mode) | 24 (15 men, 9 women)<br>Age: 35 ± 12 yr<br>Body mass, 71.4 ± 13.0 kg<br>Height: 1.72 ± 0.08 m<br>Body mass index: 24.9 ± 4.1 kg/m$^2$ |
| Real-world outdoor experiment | A diverse group of healthy adults performed naturalistic outdoor activities. | Naturalistic walking for 20 bouts at varying speeds.<br>Steady walking at a self-selected pace.<br>Steady running at a self-selected pace. | Activity-specific smartwatch (Apple Watch Series 1, activity specific mode)<br>Smartwatch (Fitbit Charge 4, general mode) | 30 (17 men, 13 women)<br>Age: 34 ± 14 yr<br>Body mass, 71.4 ± 13.0 kg<br>Height: 1.72 ± 0.08 m<br>Body mass index: 24.1 ± 3.5 kg/m$^2$ |

**Supplementary Table 2.** Linear mixed model analysis of age, sex, and BMI effects on energy expenditure estimation for each method across three outdoor conditions: real-world walking, outdoor walking, and outdoor running (*n*=30).

| **Method** | **Term** | **Effect size** | **95% CI Lower** | **95% CI Upper** | ***p*-value** |
|---|---|---|---|---|---|
| Activity-specific smartwatch | Age | 0.003 | -0.166 | 0.173 | 0.971 |
| | Sex | 0.007 | -4.750 | 4.760 | 0.998 |
| | BMI | -1.5e-5 | -7.9e-5 | 5.9e-5 | 0.755 |
| Generic Smartwatch | Age | -0.186 | -0.495 | 0.123 | 0.238 |
| | Sex | -5.050 | -13.700 | 3.630 | 0.254 |
| | BMI | -1.5e-5 | -1.41e-4 | 1.11e-4 | 0.814 |
| Heart rate model | Age | 0.276 | -0.157 | 0.709 | 0.211 |
| | Sex | 0.250 | -11.900 | 12.400 | 0.968 |
| | BMI | -2.7e-5 | -2.03e-4 | 1.50e-4 | 0.768 |

BMI, body mass index; CI, confidence interval.

**Supplementary Figure 1.** Bland-Altman analysis across methods and study conditions. Differences between estimated and ground-truth respirometry energy expenditure are shown for the activity-specific smartwatch (Apple Watch, left column), generic smartwatch (Fitbit, middle column), and heart rate model (right column) during real-world walking (top row), outdoor walking (middle row), and outdoor running (bottom row). Each point represents one participant. The dotted line indicates bias, dashed lines indicate the 95% limits of agreement (LoA).

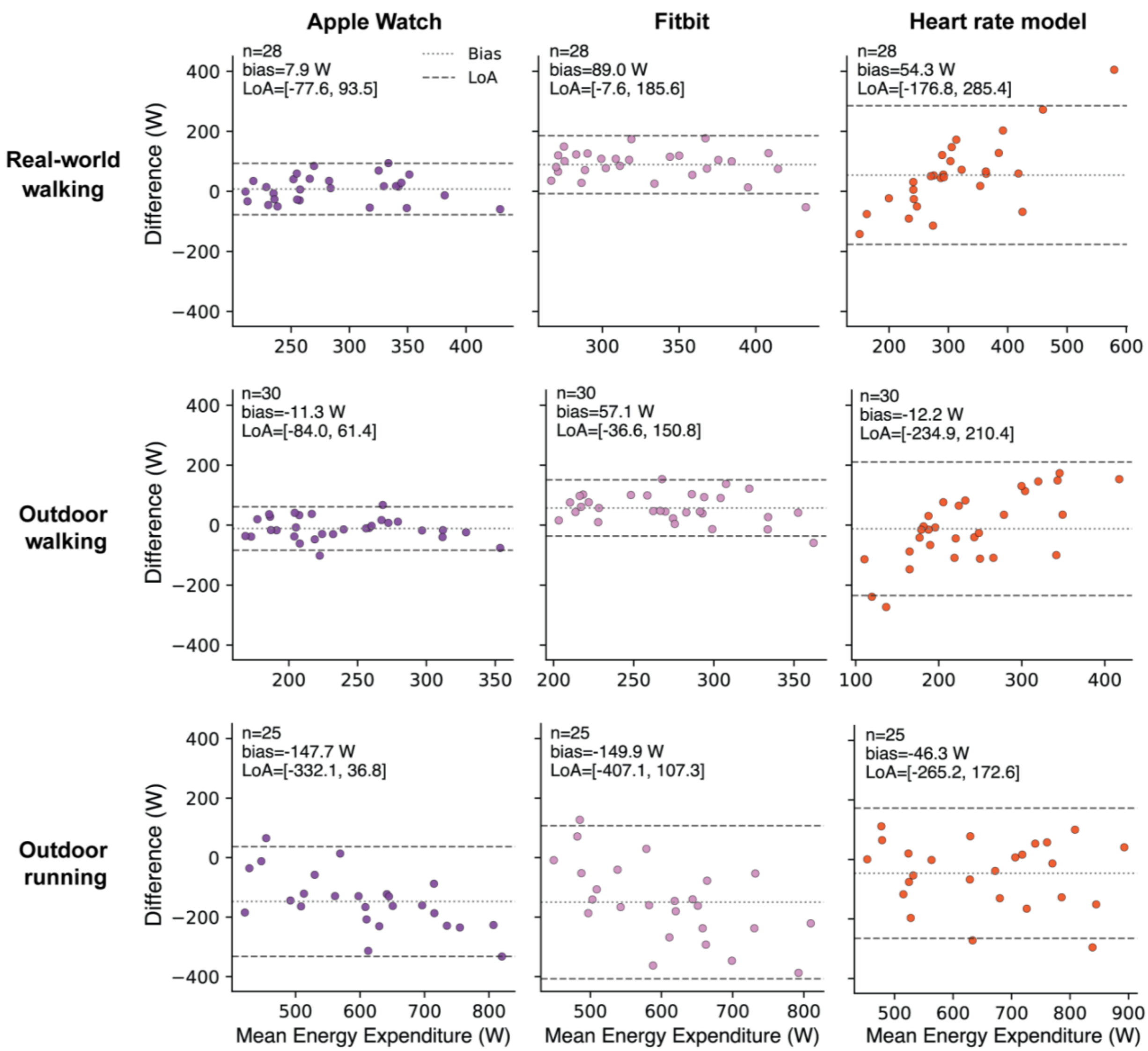

Supplement: Supplementary file 1 [file Datasheet1.pdf]
